# Achieving the potential of mHealth in medicine requires challenging the ethos of care delivery

**DOI:** 10.1017/S1463423622000068

**Published:** 2022-03-22

**Authors:** John P. Ratanawong, John A. Naslund, Jude P. Mikal, Stuart W. Grande

**Affiliations:** 1 University of Minnesota, School of Public Health, Division of Health Policy and Management, Minneapolis, MN, USA; 2 Harvard Medical School, Department of Global Health and Social Medicine, Boston, MA, USA

**Keywords:** implementation, innovation culture, medical ethos, mHealth, mobile health applications

## Abstract

Mobile Health (mHealth) interventions have received a mix of praise and excitement, as well as caution and even opposition over recent decades. While the rapid adoption of mHealth solutions due to the COVID-19 pandemic has weakened resistance to integrating these digital approaches into practice and generated renewed interest, the increased reliance on mHealth signals a need for optimizing development and implementation. Despite an historically innovation-resistant medical ethos, mHealth is becoming a normalized supplement to clinical practice, highlighting increased demand. Reaching the full potential of mHealth requires new thinking and investment. The current challenge to broaden mHealth adoption and to ensure equity in access may be overcoming a “design purgatory,” where innovation fails to connect to practice. We recommend leveraging the opportunity presented by the COVID-19 pandemic to disrupt routine practice and with a new focus on theory-driven replicability of mHealth tools and strategies aimed at medical education and professional organizations.

## Introduction

The promise of Mobile Health (mHealth) is a paradox of opportunity and risk. Digital health technologies have fundamentally transformed the way we interact with each other, access health information, and seek care. As an example, during the COVID-19 pandemic, mHealth technologies have shown their effectiveness in supporting usual services disrupted due to the pandemic and restrictions on in-person care. Further, mHealth has been helpful in monitoring atrial fibrillation, predicting COVID-19 symptom progression, and identifying need for ventilation (Adans-Dester *et al*., 2020; Linz *et al.*, 2020). Yet, as these technologies continue to develop with mixed evidence supporting its effectiveness, mHealth still faces design challenges, a skeptical medical community, deeper issues of low impact and retention, and serious equity gaps (Bhattacherjee and Hikmet, 2007; Marcolino *et al.*, 2018; Safavi *et al.*, 2019). Notably, COVID-19 has exposed and exacerbated major gaps in access/use of digital health tools among marginalized Black and Hispanic populations (Uscher-Pines *et al.*, 2021). Together, these limit the widespread adoption and sustainability of digital platforms in primary care and medicine (Steinhubl *et al*., 2015; Gagnon *et al.*, 2016; Marcolino *et al.*, 2018; Bally and Cesuroglu, 2020).

mHealth has seen exponential growth with new technologies, their rapid development, and burgeoning integration across health care (Steinhubl *et al*., 2015). From smartphone applications to wearable devices, volumes of personal data can be tracked, yielding new insights about our behaviors, physiological states, and risk of disease (Steinhubl *et al.*, 2015). The allure of these technologies and their rapid innovation have promised to improve care by empowering individuals with new opportunities to self-manage disease, engage in health-promoting activities, make informed decisions, and get the right treatment at the right time (Knight *et al*., 2014; Floch *et al.*, 2018; Grande *et al.*, 2019). This unprecedented engagement, while exciting, should also raises concerns over the quality of the evidence behind these technologies and their implications for patient–provider relationships.

The development of mHealth technologies has in some cases outpaced rigorous research on effectiveness (Steinhubl *et al*., 2015); particularly, the efficacy of new tools in the hands of consumers and care providers in real-world settings remains uncertain (Marcolino *et al.*, 2018; Steinhubl and Topol, 2018). Evaluations of existing mHealth tools show mixed evidence of improved care delivery (Sahin, 2018; Huckvale *et al.,* 2019). High attrition rates, a lack of sustained outcomes, and limited generalizability – particularly in marginalized groups – are often reported in digital health research, leading many to suggest that the problem with mHealth lies in its development (van Heerden *et al*., 2012; Matthew-Maich *et al.*, 2016; Shaw *et al.*, 2017; Stowell *et al.*, 2018; Druce *et al*., 2019).

Following the emergence of the COVID-19 pandemic the speed and expansion of new mHealth tools have been unprecedented, calling attention to both a rising interest in mHealth and a need to assess the quality of these efforts (Kondylakis *et al.,* 2020). One review of new mHealth tools identified psychological distress owing to the pandemic as a primary target of mHealth interventions, recognizing opportunistic gaps in the delivery of mental health care (Zhang and Smith, 2020). Another review pointed to the potential benefits of mHealth to supplement patient health communication, support clinical consultations, and reduce feelings of social isolation during the pandemic, yet there were also concerns related to study quality (Kondylakis *et al.,* 2020). As COVID-19 expands the demand for mHealth, which will likely persist as many aspects of care delivery will continue to rely on remote technologies in the months and years ahead, this may be ideal timing to consider how to refine and optimize mHealth to ensure its sustainability as an effective intervention rather than as a short-lived innovation.

In lower resourced countries, where much has been reported on the use of mHealth to meet growing demand, reports identify new and emerging digital health technologies that show limitations of mHealth adoption, sustainability, and patient retention (Labrique *et al.*, 2013; Bhatia *et al.* 2020). Similar challenges have also been observed in low-income communities in the USA (Nouri *et al.* 2020). When combined with the limited use of health behavior frameworks or underlying theories to guide the work, mHealth’s attrition issues, gaps in access, and muted impact raise important questions. How have researchers designed these studies and have they meaningfully engaged target users? What is causing high rates of attrition? How do we evaluate the overall health impact of these tools? Or what types of mHealth tools are the most effective for specific conditions or within specific populations? Considering the extensive interest and growing commentary on how digital technologies are poised to improve patient care following the COVID-19 pandemic, there is a paucity of evidence on ways to integrate the two. Recognizing this gap, this paper considers opportunities to address compatibility concerns between mHealth and current systems with the hope of informing design/implementation of mHealth tools in routine care settings.

## Methods

This brief report considers the promises and perils of mHealth interventions through an intentional examination and review of published literature. Sources were selected from search engines like Google Scholar, PubMed, EBSCOhost, and OVID. Boolean search terms and operators organized a strategy to include the following: digital*, mHealth*, intervent*, implement*, medicine, primary care, design*, mobile, and health. Reference sections of key articles with high relevance to the goal of this paper were reviewed for additional sources. Published date was not an applied criterion and priority was given to more recent articles and those with highest degree of topical relevance. Our assessment of mHealth included a broad exploration of findings drawn from commentaries, editorials, literature reviews, implementation studies, and primary research. As this review was intended to consider drawbacks and advantages of mHealth and propose possible solutions to advance the field, the process of including and excluding studies was contingent on relevance to the underlying discussion – the promises and perils of mHealth.

## Results

Our review of current mHealth trends reflects a renewed interest and need for innovation in medicine. We further observe that health system responses to the COVID-19 pandemic offer an opportunity to challenge the readiness of primary care or health care to adopt mHealth solutions.

### Promise of mHealth

Despite significant issues in applicability and effectiveness, mHealth technologies are continually praised for their innovation and for promoting patient engagement in health care (Rowland *et al.,* 2020). In many ways this praise is warranted, where mHealth interventions have created new avenues for delivering care, allowing providers to reach patients in ways previously unattainable (Graffingna *et al.*, 2016). Developers envision their platforms as innovative, suggesting their adoption not only crosses geographical and temporal barriers but leads to greater efficiency, communication between users, health outcomes, and accessibility to care (Steinhubl *et al.*, 2015; Gagnon *et al.*, 2016). Additionally, these technologies have allowed patients to access more information about their own health than ever before.

This is demonstrated in the iMHere System study by Parmanto *et al.* (2013), where spina bifida patients could see and report mood-related symptoms to their providers under a mobile mental health app. The study engaged patients to prioritize relevant app features that included bi-directional communication and self-care, which translated to higher adherence and self-care practices (Parmanto *et al.*, 2013). Among health workers a recent review from low- and middle-income countries showed how mHealth helped care coordination, how it helped streamline workflow and feedback, and how it improved care and relationships with community members (Ming *et al.* 2020; Odendaal *et al.*, 2020). Under COVID-19, mHealth has emerged as a prime strategy for providing continuous health care, while enabling patients to maintain social distance and avoid unnecessary exposures by offering telehealth visits, consultations, contact tracing, and home monitoring of symptoms, based on patient need. By placing power and knowledge in the hands of individuals, mHealth brings a new emphasis on patient autonomy, enhancing the way care is provided (Schmietow and Marckmann, 2019).

### Peril of mHealth

Despite the potential for mHealth to promote innovative solutions and increased access to services, the design and evaluation of mHealth interventions have been limited to stand-alone studies, with limited time horizons, that limit our ability to find sustainable solutions. Consequently, questions remain about the benefits of mHealth tools as a viable and robust care delivery platform (Free *et al.*, 2013; Marcolino *et al.*, 2018). Growing concerns among clinicians about the interoperability of these tools and their integration across older systems suggest more needs to be done to incorporate clinician workflows (Sezgin *et al*., 2018; Gruson, 2021). Many seem skeptical of how these tools enhance care and perceive ease of use as a complicating factor (Gagnon *et al.*, 2016). Among the largest digital health companies where investments and expectations are highest (Safavi *et al.,* 2019) these issues, if unaddressed, threaten the viability of future mHealth investment.

A major limitation of current mHealth design is the limited use of guiding frameworks like evidence-based theory (Salwen-Deremer *et al.,* 2020) or shared decision making (Rahimi *et al*., 2017). One review found that nearly 75% of mHealth studies lack theoretical integration into their intervention design (Buhi *et al.*, 2013). While there are several studies integrating theory to understand the mechanisms behind digital platforms and user behavior, mHealth largely sees limited application of theory despite its importance (Rowland *et al.,* 2020).

Without theory to guide design and evaluation of these needed innovations, the sustainability and efficacy of new mHealth platforms are threatened (Riley *et al.*, 2011). Efforts like person-centered design, usability testing, and ethnography are cited as clear examples of how this functionality can be managed (Huckvale *et al.*, 2019). Others offer an alternative solution by combining emerging design and evaluation practices to address more real-world patterns of behavior – particularly around inequities in access (Wilhide III, *et al*., 2016). Notably, COVID-19 has raised serious equity concerns about access/use of mMealth among the most vulnerable and marginalized including racialized groups as well as low-literacy and low-income populations (Uscher-Pines *et al.*, 2021). Regardless, the field is characterized by patch work application of varied and inconsistent behavioral theories, resulting in a robust set of pilot studies isolated on islands of knowledge rather than coalescing into a single, integrated framework.

**Figure 1.**
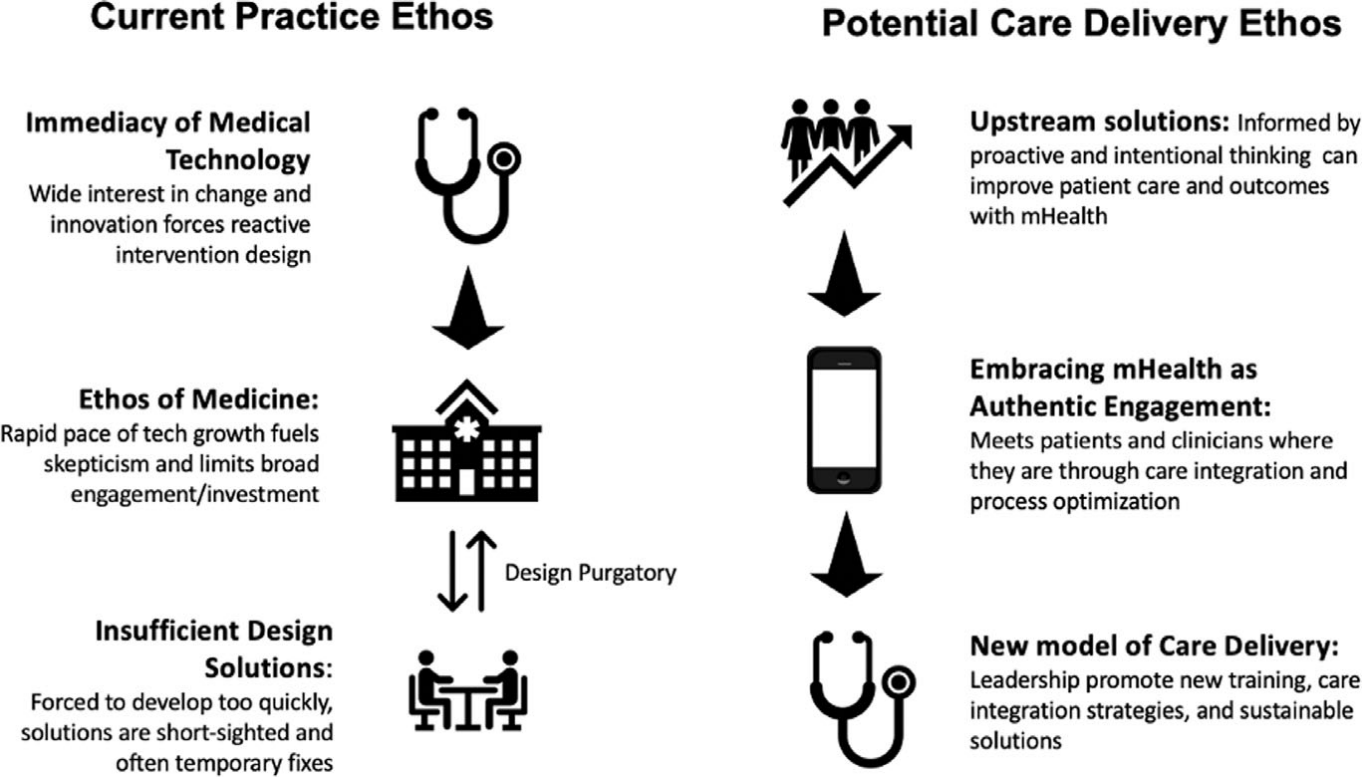
Current and potential care delivery ethos toward novel digital health platforms

Early digital health implementation had been plagued a kind of purgatory with high numbers of small underpowered studies that struggled to demonstrate clinical effectiveness outside specific populations (Figure 1) (Huang *et al*., 2017). More recent evidence shows modest improvement with acceptability, and yet problems persist with engagement, retention, and low impact of interventions (Safavi *et al.*, 2019). One meta-analysis that showed benefit of mHealth on multiple conditions including weight management, asthma, and gestational diabetes control in pregnant women (Chan and Chen, 2019) also showed limitations on participant use, cost, and “credibility” of app content. Other review evidence identifies a high degree of heterogeneity among mHealth interventions, which pose empirical issues when trying to control for effect size (Buneviciene *et al.*, 2021). Emerging data from programs in low- and middle-income countries point to simplicity, a need for partnership with care providers, and integration into compatible care ecosystems as innovative solutions to impact community engagement (O’Donnell, 2020).

One example of a tool, mWellcare, that was developed to support chronic illness management in a low-resource setting showed that partnership between clinics, technologists, and field clinicians contributed to more effective design and patient use (Jindal *et al.*, 2018). Even with examples, widespread within mHealth are independent platforms each designed with a single purpose for a specific condition (van Heerden *et al.*, 2012; Gagnon *et al.*, 2016). A lack of integration of platforms into routine practice also signals failed stakeholder engagement between information systems and primary care managers (Bally and Cesuroglu, 2020). Competing interests and siloed systems limit generalizability and applicability to real-world health systems. It stands to reason that a failure to integrate mHealth into health systems is more than a need to demonstrate effectiveness. And among patients, digital health research shows high attrition rates (Druce *et al.*, 2019), a finding supported by rates between 80% and 98% on eHealth dropout (Lie *et al.*, 2017; Meyerowitz-Katz *et al.,* 2020). Ultimately, end-users, be they patients, clinicians, or investors, need to experience the value in this technology and how to use it, which is why utilizing a design and evaluation framework for adoption is so important.

### Design and evaluation solutions

The lack of frameworks to shape more adaptive platform design solutions is a critical barrier to sustainability, yet they are one side of the problem. Fixing them alone will not be enough for mHealth to achieve its promise. Arguably, most behavior change frameworks needed to guide system integration and focus on individual behaviors rather than communication patterns between patients and providers (Salwen-Deremer *et al.*, 2020; Walsh and Groarke, 2019). Individual Behavior Change theories that use predictive factors like “having the confidence to perform an action” (Social Cognitive Theory) or “attitude related to the intention to act” (The Reasoned Action Approach) are highly context specific and are not linked to the use of digital devices. Therefore, more dynamic health behavior theories that account for the distinct features of digital health technology and behavior change theory are needed, like the Fogg Behavior Model or the Ritterband Internet Intervention Model (Riley *et al.*, 2011; Salwen-Deremer *et al*., 2020). These models appear to link behavior change principles with specific illness conditions and applications (Walsh and Groarke, 2019).

In medicine, patient complexity and co-morbidity present serious theoretical challenges to mHealth. Questions about application and feasibility within daily practice will remain; therefore, it is unlikely theoretical integration alone will solve issues of sustainability. And while theory is essential for behavior change, the disconnect between mHealth and traditional views on digital health solutions requires a shift in mindset upstream of platform development. Necessary innovations here may include working upstream, with patients and clinicians, to determine fit and applicability prior to implementation. This also necessitates a critical step for cultural adaption of mHealth tools factoring in language and other contexts applicable to specific underserved groups and populations.

The greatest shortcoming of mHealth may be a function of relevance and value for patients (Rowland *et al.,* 2020). New technologies have been designed to address recognized inefficiencies, like long wait-times or loss to follow-up, by mitigating care fragmentation using better communication between systems and users. However, merely inserting these platforms into inefficient care systems fails to achieve desired outcomes (Steinhubl *et al*., 2015). Recognizing various infrastructure limitations and a need to design integrated workflows between platforms and mHealth as well as between patient engagement and clinical app use are also critical for future solutions (Bechtel *et al.*, 2021). Systems of care behind these innovations are not adapting to accommodate them and are thus inevitably repeating prescribed models of care and habituated communication patterns between providers and patients (Lupton, 2015). Recent mHealth developments to mitigate COVID-19 reflect these retroactive or just-in-time solutions, and while they appear helpful, the basic premise of care delivery remains unchanged (Zhang and Smith, 2020). Thus, with many mHealth tools designed to retroactively fit into current care models, it is likely that the underlying issues that necessitated a digital health solution will persist.

### Current and potential care delivery ethos toward novel digital health platforms

#### Ethos of care delivery

Inside the traditional ethos of care delivery is an underlying incompatibility between the current health care system and digital health technologies. One area where tension is most acute is at the point of care. Here, where patients are most vulnerable and clinicians feel most pressured, there is a low tolerance for new strategies. In this way, mHealth presents an opportunity to challenge anachronistic models of care by offering new modes of care delivery. Innovative models that prioritize patient empowerment and patient-centered decision making should be viewed as a positive disruption to traditional care delivery strategies. Given the current medical ethos or culture, inherent intransigence potentially stymies benefit that mHealth could offer. In effect, mHealth adoption will muddle along unless the culture of medicine adopts a new vision of care delivery. Further, if these platforms are integrated without the full participation and intentionality of health care systems, there is an expressed fear for leaving some patients behind (Lyles *et al.*, 2021). Arguably, this shift has already begun. Studies over the past several months indicate that the COVID-19 pandemic has fundamentally altered how health care views digital technologies and their application in routine practice (Rowland *et al.,* 2020).

At the system level, innovative digital technologies have been viewed with much skepticism, with many seeing their presence disrupting traditional care delivery. Understandably, any innovation bringing change to the status quo is met with a level of uncertainty and resistance; sticking with what we know is natural, and skepticism of the new is rational (Jacob *et al*., 2020). However, current perceptions on digital innovation and mHealth struggle to see value in the potential benefits of these interventions in the real world (Ostrovsky and Barnett, 2014). For instance, a study by Gagnon *et al.* (2016) explored the barriers and facilitators of mHealth adoption by health care professionals and found perceived usefulness, costs, disturbed workflow, interoperability, and security issues as concerns among providers. Certain perceived challenges such as these can make or break an intervention’s longevity (Bhattacherjee and Hikmet, 2007; Pati *et al.*, 2013; Steinhubl *et al.*, 2015). Even so, many clinicians and health system administrators have been innovation averse, leaving some systems under-equipped to maximize the benefits of novel technologies in routine clinical practice and service delivery. Likely, a lack of investment in innovation labs or internal design workshops may have contributed to perceptions of mHealth’s limited utility in practice. Curiously, as the COVID-19 pandemic shuttered facilities and rendered traditional delivery systems ineffective, recent investments into innovation labs and the rapid and global implementation of mHealth tools to meet patient demand signal a major shift in health systems’ interest in digital health.

Until recently, the traditional medical ethos had inhibited mHealth platform integration. As the COVID-19 pandemic forced health systems to rethink older models of care, mHealth tools have emerged as a promising solution. This dramatic shift in thinking has exposed a generational opportunity to leverage these technologies and collective action to shape, not retrofit, the future of health care (Figure 1).

## Discussion

In this paper, we contend that the COVID-19 pandemic represents a turning point in a systems-wide willingness to engage with the unique opportunities of mHealth – but without theory-driven design and increased efforts to support equitable access – aligned with use of theory and design of platforms from the outset – we risk repackaging old inequities in new media.

### Upstream solutions

App developers and researchers have an opportunity presented by the COVID-19 pandemic to challenge the traditional ethos of care delivery. Upstream solutions, like mHealth integration into medical education or health system-based design labs, that promote replicability not just innovation will likely change the science of mHealth and build a more robust evidence base (Table 1). Further, strong science demands new models and platforms for engaging patients get tested and retested to ensure reliability and replicability, and sustained delivery. There is an opportunity for leading academic medical centers (AMCs) to embrace mHealth as a legitimate model of care delivery and use top-down organizational commitment to promote its integration. This challenge to the ethos of care delivery will increase demand for stronger theory-driven mHealth within medical and health professions and ensure sustainability.

**Table 1.** Potential upstream solutions to address care delivery ethos

I. Incorporate clinical innovation training programs into academic medical and other health professions education. Example: “Accelerating Change: Fostering Innovation in Healthcare Delivery at Academic Medical Centers” by Ostrovsky and Barnett (2014). Authors outline two initiatives that promote innovation training in academic medical education: (1) an institutional innovation incubator program would function to facilitate the development of new care delivery products or services for patients and providers. These incubators included personnel experienced in entrepreneurship, software development, financing, legal advising, and those in innovation network tracks, all of which would support clinician innovators. Several innovation incubators at major academic medical centers are also cited. (2) a clinician-innovator career track that would allow training clinicians to develop projects that lead to new innovations and products.
II. Utilize academic medical centers (AMC’s) as settings for new mHealth design and implementation studies. Example: “Building Digital Innovation Capacity at a Large Academic Medical Center” by Mann *et al*. (2019). This case study by Mann *et al.* reviews the formation and evaluation of the Digital DesignLab at NYU Langone Medical Center. The initiative consisted of a cross-functional team of experienced faculty with backgrounds in academia, clinical practice, and digital solution fields. Digital Design Lab developed a selection process for new digital health innovations projects that are likely to see practice integration. The initiative served as an AMC-centralized process for evaluating and supporting projects trying to develop novel digital health solutions.
III. Promote innovation culture and adoption of novel digital health technology through top-down leadership within medical academia. Example: “Tradition Meets Innovation: Transforming Academic Medical Culture at the University of Pennsylvania’s Perelman School of Medicine” by Pati *et al*. (2013). Guided by principles from business transformation models and workforce environment innovation, the Perelman School of Medicine at the University of Pennsylvania implemented the NIH-TAC (Transforming Academic Culture) in 2009. The purpose of this initiative was to drive institutional culture changes within academic medicine by faculty leadership to foster more innovative career paths and programs that align with the rapidly increasing demands for innovation in medicine.

Changing ethos requires socializing clinicians and frontline health workers to the benefits of mHealth. Creating greater exposure to evidence-based innovation design practices early, researchers can work with clinicians and health workers to integrate these tools. Such co-design practices and exposure to human-centered design practices could also help open perspectives to transformative innovations (Ostrovsky and Barnett, 2014) and support efforts to expand the reach of these digital platforms in particular to underserved and vulnerable patient groups. Clinical innovation training that complements existing curriculum may also provide the necessary exposure to improve the utilization of future mHealth platforms (Pati *et al.*, 2013; Ostrovsky and Barnett, 2014). Moreover, AMCs could be “ground zero” for mHealth adoption and fomenting culture change. With strong commitment to novel platforms, these settings could lead by demonstrating mHealth’s utility. Additionally, a culture shift encouraged by leadership within academic medicine could lead to greater recognition and acceptance of digital health innovation.

The advent of these digital technologies may guide primary care and medicine into the future, but until this opportunity presented by COVID-19 is thoughtfully exploited, mHealth will remain on the margins, or an extension of health care services destined to perpetuate existing inequities in access and quality. As patients and clinicians increasingly experience the benefits of mHealth, demand will surely increase for both trusted tools and clinicians who use them and use them well. As criticism of mHealth has rightly focused on issues of generalizability, inequitable access, safety, and the need for integration of behavioral theory, the current ethos within medicine lacks inspiration to act. There is an opportunity here to leverage both upstream (culture) and downstream (design) insights to further the promise of mHealth. Moving from theory to practice, mHealth requires the intentional engagement of patients, clinicians, health system administrators, and leaders in medicine to think beyond traditional perspectives that may resist change. It requires digital health proponents and critics to recognize that technology can only improve care by moving toward replicability, reaching vulnerable patient groups who stand to benefit most from improved access to quality and timely care, and novel approaches to ensure sustained adoption and delivery of these digital platforms in routine care settings. This ensures mHealth’s future as a respectable and appropriate care delivery solution and not as an ad hoc supplement to existing medical care.
